# Co-development and Usability Testing of Research 101: A Patient-Oriented Research Curriculum in Child Health (PORCCH) E-Learning Module for Patients and Families

**DOI:** 10.3389/fped.2022.849959

**Published:** 2022-07-06

**Authors:** Catharine M. Walsh, Nicola L. Jones, Graham A. McCreath, Veronik Connan, Linda Pires, Autumn Q. H. Chen, Aliza Karoly, Colin Macarthur

**Affiliations:** ^1^Division of Gastroenterology, Hepatology and Nutrition and the SickKids Research and Learning Institutes, The Hospital for Sick Children, Department of Paediatrics and the Wilson Centre, Temerty Faculty of Medicine, University of Toronto, Toronto, ON, Canada; ^2^Division of Gastroenterology, Hepatology and Nutrition and the SickKids Research Institute, The Hospital for Sick Children, Department of Paediatrics and Department of Physiology, University of Toronto, Toronto, ON, Canada; ^3^SickKids Research Institute, The Hospital for Sick Children, Toronto, ON, Canada; ^4^Department of Clinical Dietetics, The Hospital for Sick Children, Toronto, ON, Canada; ^5^Canadian Child Health Clinician Scientist Program, Toronto, ON, Canada; ^6^The Hospital for Sick Children, Toronto, ON, Canada; ^7^SickKids Research Institute, The Hospital for Sick Children, Department of Paediatrics, University of Toronto, Toronto, ON, Canada

**Keywords:** patient-oriented research, patient engagement, capacity development, child health research, online education

## Abstract

**Background:**

Engaging patients and families as research partners increases the relevance, quality, and impact of child health research. However, those interested in research engagement may feel underequipped to meaningfully partner. We sought to co-develop an online learning (e-learning) module, “Research 101,” to support capacity-development in patient-oriented child health research amongst patients and families.

**Methods:**

Module co-development was co-led by a parent and researcher, with guidance from a diverse, multi-stakeholder steering committee. A mixed-methods usability testing approach, with three iterative cycles of semi-structured interviews, observations, and questionnaires, was used to refine and evaluate the e-learning module. Module feedback was collected during testing and a post-module interview, and with the validated System Usability Scale (SUS), and satisfaction, knowledge, and self-efficacy questionnaires. Transcripts and field notes were analyzed through team discussion and thematic coding to inform module revisions.

**Results:**

Thirty participants fully tested Research 101, and another 15 completed confirmatory usability testing (32 caregivers, 6 patients, and 7 clinician-researchers). Module modifications pertaining to learner-centered design, content, aesthetic design, and learner experience were made in each cycle. SUS scores indicated the overall usability of the final version was “excellent.” Participants' knowledge of patient-oriented research and self-efficacy to engage in research improved significantly after completing Research 101 (*p* < 0.01).

**Conclusions:**

Co-development and usability testing facilitated the creation of an engaging and effective resource to support the scaling up of patient-oriented child health research capacity. The methods and findings of this study may help guide the integration of co-development and usability testing in creating similar resources.

## Introduction

Patient engagement in health research, currently a dominant discourse in North America (1, 2) and around the world (3), refers to the involvement of patients and communities as equal research partners, as opposed to participants, in the design and conduct of research. Patient-oriented research in Canada is defined as research that engages patients as equal partners, focuses on patient-identified priorities, and aims to improve patient outcomes (1). There is growing evidence that patient engagement in research encourages equity, improves outcome selection, facilitates participant recruitment and retention, increases the quality, credibility, and applicability of evidence, and facilitates knowledge translation (4–7). In pediatrics, partnering with children and their caregivers in research aligns with the principles of child and family-centered care (8) and can leverage rich insights and expertise about child health that may not otherwise be captured (9–11). For engagement to be successful, training for all stakeholders, based on best practices in education pedagogy, is recommended (5, 12, 13). In particular, patients and families require the knowledge and skills to be authentic research partners, and researchers and healthcare professionals must appreciate the added value of patient partners and understand how to collaborate effectively (14, 15).

Insufficient preparation and training are associated with patient and family partners feeling unable to contribute (16–19), lacking an understanding of research methodology and associated technical language (17, 20–22), and misunderstanding their role (21, 23–25). In the context of research involving adults, patient partners reported improved knowledge of research (24, 26–28) and study content (26, 29) after training. However, training resources and empirical work describing their development and effectiveness are limited. A survey of young persons' advisory groups from 7 countries identified a dearth of appropriate training materials as a major barrier to engagement (30).

To address the need for pediatric-specific education, we created the Patient-Oriented Research Curriculum in Child Health (PORCCH), an open-access online curriculum with specialized modules for different stakeholder groups. The goal of PORCCH is to build capacity in patient-oriented child health research and support meaningful and authentic patient (and other stakeholder) partnership in health research (31). Online learning (e-learning) has several advantages, including wide dissemination, remote and asynchronous learning, and flexibility for learners to customize their education (32–35). Central to the quality and evaluation of e-learning materials is their usability, which refers to the effectiveness, efficiency, and satisfaction with which users can achieve a specific set of tasks in a particular environment (36). The PORCCH curriculum was co-developed through a sharing of power and responsibility across all stages between clinicians, researchers, patients and families, and other stakeholders. This level of engagement is equivalent to what INVOLVE in the United Kingdom and the International Association for Public Participation (IAP2) refer to as co-production and collaboration, respectively (37, 38). The aims of this study were to (1) co-develop, “Research 101,” the PORCCH module intended to strengthen capacity in patient-oriented child health research among patients and families and other stakeholders without a formal background in research, (2) refine module content through iterative usability testing, and (3) evaluate the impact of the module on self-efficacy and knowledge. The PORCCH curriculum also includes two additional modules, namely “Patient Engagement 101” that focuses on engaging patients and families in health research and “Research Ethics 101,” which focuses on general principles of research ethics and ethical issues specific to patient-oriented research.

## Methods

### Co-development of Research 101

The Ontario Strategy for Patient-Oriented Research (SPOR) SUPPORT Unit—part of the Canadian Institutes of Health Research (CIHR) Strategy for Patient-Oriented Research—put out a call in 2016 for novel online training materials to build capacity in patient-oriented research. Given the dearth of child-focused online curricula on patient-oriented research, a collaborative group of clinicians, researchers, and parents submitted a successful application to this competition for the development of PORCCH. The collaborative group was multi-disciplinary and multi-site, with experience and expertise in child health.

A PORCCH steering committee, comprising two clinician-researchers, two SPOR SUPPORT Unit leads, three parent partners (one recruited from a SPOR research network and two from a hospital family advisory committee), a knowledge translation expert, an educational researcher, and two instructional design experts, was formed to provide support, advice, and guidance to the module co-leads. The parent partners on the steering committee all had lived experience with a child with a long-term health condition. In line with SPOR's guiding principles for patient engagement, a collaborative process was maintained throughout module co-development, whereby all partners worked together from the start to identify educational needs, set objectives, and co-develop the module in a manner that acknowledged and valued each other's expertise and experiential knowledge (14).

Co-development of Research 101 was co-led in equal partnership by a parent (AK) and researcher (CM). The principal aim guiding module co-development was to meet the training needs of stakeholders unfamiliar with health research. The initial process involved bringing together patients and families, clinicians, researchers, and knowledge translation experts from multiple pediatric centers in a series of in-person community consultations to identify relevant health research concepts and content to be included in the module. Next, module co-leads were responsible for collating and reviewing the relevant patient-oriented research literature and pertinent content identified through initial consultations with key stakeholders. This was then reviewed in collaboration with the steering committee to inform module content.

To plan out the module, a storyboard was created, which was iteratively reviewed by the steering committee and revised by the module co-leads six times over 9 months. After the storyboard was finalized, a module prototype was programmed using Storyline 360 (Articulate Global Inc., New York). The prototype was created in accordance with Mayer's principles of multimedia design (39) and best practices in plain language writing, including short sentences, simple words, identification of all acronyms, positive tone, active writing style, and large text (40). Additionally, the readability of the module was targeted at a grade 6 reading level and content was designed to meet the Web Content Accessibility Guidelines (WCAG) 2.0 Level AA requirements (e.g., captions, minimum visual contrast ratio) designed to ensure web-based content is perceivable, understandable, navigable, and interactive (41). As before, module prototypes were internally reviewed and approved by the steering committee. Module co-leads met monthly in person to discuss, draft, and revise module content and layout. Over a 6-month period, the steering committee met three times with the co-leads to discuss and provide feedback on module content and design. The module co-development process is outlined in Supplementary Appendix 1.

This process culminated in an e-learning module prototype, “Research 101,” that is delivered in two thirty-minute parts. Part 1, “What is Health Research and Who is Involved?” defines patient-oriented research and describes the value of patient engagement in research, the “key players” in health research, and the difference between research participation and research partnership. Part 2, “Timeline of a Research Study,” covers the key phases of a health research study, the impact, challenges, and benefits of patient-oriented research, and how patients and families can engage as partners. Both Parts include interactive tools, video vignettes, assessment exercises, certificates of completion, and links to additional resources for further learning.

### Refinement and Evaluation of Research 101

A mixed-methods usability testing approach, with three iterative cycles of semi-structured interviews and observations, along with satisfaction, knowledge, and self-efficacy questionnaires, was used to refine and evaluate the e-learning module prototype. This user-centered design approach, which has been used previously for usability testing of online patient education and electronic healthcare apps (32, 42), is an iterative process of implementing a design, learning from discussion and thematic analysis of feedback, and making subsequent design refinements (43). The study was approved by the SickKids Research Ethics Board. This manuscript was prepared in accordance with the Guidance for Reporting Involvement of Patients and the Public (GRIPP2) reporting checklist (44).

#### Participants

English-speaking patients (10–18 years), caregivers of pediatric patients, and child health clinician-researchers were recruited across Canada via email and electronic newsletters from family advisory networks, SPOR SUPPORT Units, the Canadian Child Health Clinician Scientist Program, and CHILD-BRIGHT. Patients and caregivers, the module's intended end-users, were predominantly recruited as well as a smaller proportion of clinician-researchers, since they work directly with patients and families and may thus provide additional knowledge and perspectives on relevant training needs and issues in patient-oriented research. Individuals who expressed interest in participating were contacted by a research assistant who explained the purpose of the study and answered any questions. Participants were excluded if they had any cognitive, perceptual, or motor limitations that could restrict their ability to explore and interact with the module. Usability testing was conducted with different participants in each round to increase the diversity of perspectives sampled. A maximum variation purposive sampling approach was used to ensure the sample included individuals with diverse perspectives, specifically with respect to a wide range of familiarity with patient-oriented research and variability with respect to education level and geographic location (45, 46). Informed consent was obtained from all adult participants, and assent, or a child's verbal agreement to participate, was provided by all patient participants, in addition to consent from a legal guardian. Participants were compensated for their time with a $25 gift card (47).

#### Usability Testing

Audiotaped usability testing sessions were carried out between November 2018 and January 2020 by a research assistant (VC). Testing took place either in-person at an academic children's hospital or over the phone, according to participant preference and geographic location. Participants first completed a baseline questionnaire to collect demographic information (Supplementary Appendix 2). They then received brief instructions about the usability testing protocol. They were asked to “think aloud” as they completed the module, commenting on what they liked and disliked, any difficulties they encountered, and questions that arose. To facilitate “loud thought,” participants were asked questions to elicit their understanding of various aspects of the module (e.g., “What is the module asking you to do at this point?”) and to solicit participants' suggestions for improvement (Supplementary Appendix 3). Field notes pertaining to usability and any technical problems encountered were also recorded.

Immediately after completion of Research 101, participants engaged in a one-on-one, semi-structured interview, comprising a series of standardized open-ended questions regarding the module's usability (Supplementary Appendix 4). Probing questions were used to elicit further details. Participants were then asked to complete an e-learning satisfaction questionnaire (Supplementary Appendix 5), which our group developed and used in a similar study (32), as well as the System Usability Scale (SUS), a validated questionnaire for evaluating user satisfaction of technologies (48). The SUS contains 10 statements scored on a five-point strength of agreement scale. Raw scores are adjusted to account for positively and negatively oriented questions, and total SUS scores range from 0 to 100, with associated adjectives from “worst imaginable” to “best imaginable,” respectively (48).

Following the first testing cycle, the module prototype was refined through thematic analysis of the usability testing interviews, field notes, and questionnaires. A second usability testing cycle was then conducted to garner further recommendations for potential changes to the online curriculum. Because of a programming error unintentionally introduced in cycle 2, a third round of confirmatory usability testing, comprising only the quantitative evaluations, was conducted to confirm that all issues related to the error were resolved.

#### Evaluation of Impact (Knowledge and Self-Efficacy)

To evaluate the impact of the e-learning module on users' self-efficacy, participants completed a questionnaire—developed based on Bandura's framework for constructing self-efficacy scales—before and after viewing the module (Supplementary Appendix 6) (49). Participants also completed a multiple-choice test to measure their baseline understanding of research (Supplementary Appendix 7) and rated their pre-module knowledge of patient-oriented research using a five-point Likert scale. The knowledge test was designed to assess the “knows how” level of Miller's pyramid (50) and was pilot tested on 6 individuals (3 patients/families, 3 researchers/clinicians/trainees) to ensure clarity and readability. After module completion, the knowledge test and self-rating were re-administered to determine knowledge acquisition.

#### Sample Size

A sample size of 15 participants per usability testing cycle was selected; a sample size considered adequate from a usability testing perspective to ensure thematic saturation (51, 52).

#### Data Analysis

##### Usability Testing

Audiotaped usability testing sessions and post-module interviews were transcribed verbatim, de-identified, and imported into Dedoose (SocioCultural Research Consultants; Los Angeles, California), a mixed-methods data analysis program, to facilitate data organization and analysis. Coding was both deductive, with published usability attributes (53–56) informing development of an initial coding scheme, and inductive, to allow other codes not initially anticipated to emerge. After each usability cycle, two coders (GAM, AQHC) individually read the transcripts and field notes to identify preliminary codes with regard to usability (e.g., satisfaction, efficiency, learnability, errors) and then refined them through systematic iterative coding and sorting using the constant comparison method (57, 58). Codes were then grouped into usability-related themes and subthemes (59), using published frameworks from the usability literature (53, 60–62) and a previous e-learning usability study (32) as sensitizing concepts. The final coding framework, presented in Supplementary Appendix 8, was then applied to all transcripts. Disagreements or uncertainties between coders were resolved through discussion and consensus with a third coder (CMW). The study team met regularly to discuss and refine the evolving themes and coding framework, and used their expertise in education, child health, and research to help clarify and critique the findings. Thematic saturation was monitored through the number and novelty of the usability issues raised by subsequent testers, both within and across usability testing cycles (58). To enhance trustworthiness of the findings (63), reflexivity was employed and the team, including parents, clinicians, and non-clinician researchers, questioned and challenged each other's assumptions throughout the analysis. This process, which enabled critical reflection, examination, and exploration of the research process from different positions, informed the team's reflections on patient engagement.

##### Evaluation of Impact (Knowledge and Self-Efficacy)

Data from the demographic, satisfaction, self-efficacy, and knowledge questionnaires were summarized using means and standard deviations for continuous variables and with counts and proportions for discrete variables.

Pre-post changes in self-efficacy and knowledge were evaluated with paired *t*-tests (α = 0.05, two-sided). Quantitative analyses were conducted in R version 4.0.0 (R Core Team; Vienna, Austria).

## Results

### Participant Characteristics

Thirty-two caregivers, 6 patients, and 7 child health clinician-researchers participated (15 in each round), more than half of whom had not previously engaged in patient-oriented child health research. Participant characteristics for each usability round are shown in Table 1. Module completion time (±SD) was similar between usability cycles, with a mean duration of 58 ± 10 min in cycle 1, and 62 ± 17 min in cycle 2.

**Table 1:** Participant characteristics.

**Characteristic**	**Cycle 1 (*n* = 15)**	**Cycle 2 (*n* = 15)**	**Cycle 3 (*n* = 15)**
**Primary role**			
Caregiver	11	11	10
Patient	2	2	2
Child health clinician-researcher	2	2	3
**Gender**			
Female	13	13	15
Male	2	2	0
**Geographical region (i.e., Canadian province)**			
Alberta	4	1	2
British Columbia	0	1	2
Manitoba	0	1	0
Nova Scotia	1	0	1
Ontario	10	10	10
Quebec	0	1	0
Saskatchewan	0	1	0
**Education**			
Elementary	2	2	0
Secondary	0	2	0
Some college or university	2	1	1
College/University	6	5	7
Masters	3	3	4
MD or PhD	2	1	3
Not specified	0	1	0
**Has previously engaged in patient-oriented child health research**			
Yes	6	7	7
No	9	8	8
**Has used e-learning before**			
Yes	13	13	13
No	2	2	2
**Comfort level using a computer** ^a^	4.60 ± 0.63	4.20 ± 1.08	4.53 ± 0.74
**Comfort level using the internet** ^a^	4.67 ± 0.49	4.20 ± 1.08	4.53 ± 0.74

a *Rated on a 1 (do not know anything about it) to 5 (know everything there is to know) Likert-type scale*.

### Research 101 E-Learning Module Usability

Qualitative analysis of usability testing sessions, used to inform iterative module refinements, centered around four themes: (1) learner-centered design, (2) content, (3) aesthetic design, and (4) learner experience. Themes were consistent across patients, caregivers, and clinician-researchers. All four themes were prevalent in cycle 1, leading to relatively major content and design revisions, whereas aesthetic design and relatively minor changes to ensure consistency in the presentation of the module predominated in cycle 2. Illustrative quotes for each theme and corresponding module changes are described below, with additional quotes and changes in Table 2.

**Table 2:** Examples of usability testing results and corresponding module changes.

**Topic**	**Cycle**	**Quote**	**Corresponding module change(s)**
**Learner-centered design**			
***Intuitive design***: the ease with which users know what to do next	C1 C2	“It said click “next” to continue but there's no “next,” it's just an arrow.” (P2, caregiver) “Pressing the “next” button is not obvious from the beginning.” (P4, caregiver) “Having to click on the video is not that obvious because it doesn't look like a video.” (P4, caregiver) “It does not look like there's a way to get out of the video, so it would be good if there was a little “X” on the top corner to close it.” (P15, clinician-researcher) “I'm not sure which buttons to click.” (P29, caregiver)	Ensured the phrasing of all prompts matched the corresponding on-screen content verbatim Automated transition of opening slide to reduce confusion Added conventional “play” icons to videos Added a button to enable users to easily exits videos at any time Added additional audiovisual prompts, where needed
**Content**			
***Quantity***: the amount of information contained in the module and/or repetition of information	C1	“I think it's the right amount. It's the information that needs to be there, without getting too involved in all of the other nitty-gritty stuff.” (P1, caregiver) “I think it was good. Part 1 was a limited amount, a solid introduction but not too much. And then part 2 was more demanding, but the opportunity to pause and to look up terms in the glossary, I think that would make the current content manageable.” (P20, caregiver)	
***Completeness***: extent to which the module content contained all desired information	C1	“I don't know if that needs to be in a module or not, but something relating to the fact that this is a new area, and researchers are learning about this too. To do this, researchers are having to work in different ways. I just think there needs to be this environment of understanding on both sides.” (P13, clinician-researcher)	Expanded challenges of patient-oriented research section to include mention that it is a new paradigm in health research that requires additional skills and knowledge of all involved stakeholders, including researchers
***Quality and trustworthiness***: the extent to which users perceive the content to be accurate and credible	C1	“I would challenge [researcher's name] on that. I think it's fair to say that patient and family engagement provides a possibility for making research better, but there is no science to say that research is better because of patient family engagement... I believe sweeping statements like those diminish the validity of patient and family engagement in research.” (P4, caregiver) “Do only researchers identify the gap in the knowledge. Don't patients also?” (P13, clinician-researcher) “That's funny, I feel like it depends on the researcher because, for instance, our parent panel and parent partners that help design our studies, they have participated in the surveys as well.” (P15, clinician-researcher)	Removed an anecdotal video that participants felt overstated the benefits of patient-oriented research Changed phrasing to include patients and families as parties that identify gaps in knowledge to inform new health research questions Updated the statement that a person cannot be both a participant and a partner in the same research study to state that generally research partners are not research participants and vice versa
***Relevance***: the relevance of the module to its intended users	C1	“I personally would want to see the potential for local and global impact. If I'm going to invest time in an area that's meaningful or of interest to me, knowing that maybe someone else with this experience down the road could be improved as a result. So just having that local impact.” (P5, caregiver)	Expanded opportunities of patient-oriented research section to include the potential for local impact
***Usefulness***: how useful the information is or who the information would be useful for	C1	“I think what would be more helpful is if you kind of give examples of qualitative and quantitative analysis so that people can recognize it when they see it no matter what it's called.” (P4, caregiver)	Improved characterization of qualitative and quantitative methods and how they differ
***Understandability***: content aspects such as readability, use of plain language, and explanation of important terminology	C1	“I really appreciated the way it was worded. I felt that you were tackling a complex set of processes but at the same time, it was done in a very basic way.” (P3, caregiver)	
***Age-appropriateness—content***: age-appropriateness as it relates to the content presented in the module	C2	“The medical language and the methods are hard to understand with all the terminology. To be for kids my son's age [10], you will have to lose some accuracy and really strip it back.” (P27, caregiver) “A child wouldn't watch it. I think a youth, 13 or 14, who's doing it because they're really interested would.” (P25, caregiver)	Will consider future adaptation for children
**Aesthetic design**		
***Features***: interactive elements of the module	C1 C2	“Now at the end, you sort of end up with the five circle icons sitting there. If you could enable users to hover over them and have the description pop up again, that might be useful just to recap what was there.” (P18, caregiver) “In Step 3 [of a research study] it talked again about qualitative and quantitative [methods]. It might be helpful to have those highlighted again with the definitions to hover over; in case you forget which is which.” (P21, caregiver)	Added tooltip “hover” feature to Step 2B: Choose the Type of Information to be Collected, so users can review content at their own pace after the slide is finished Ensured all instances of glossary terms in the module are highlighted and include definition-on-demand tooltips
***Multimedia components***: audio and visual aspects of the module	C2	“For most of it, everything [the narrator] says comes up on the screen, but there have been a couple of times when he's been talking and the text hasn't come up on the screen.” (P25, caregiver)	Ensured consistency between narration and on-screen text
***Layout***: the arrangement of text and graphics on slides	C1 C2	“I'm not sure I like the way that the writing shows up in the arrows and the circle. It's kind of small and it's on a diagonal.” (P1, caregiver) “The “click to continue” [prompt] is sitting on top of another word.” (P11, caregiver)	Ensured all text was presented horizontally and easily readable Fixed instances of prompts obscuring text
***Navigation***: the ability of the user to easily move around the module	C1	“When I go back, I don't remember where I was. So, something that identifies what you've already looked at would be useful.” (P1, caregiver)	Added checkmarks as a navigational aid to help users visually distinguish sections already viewed from sections they have yet to view
***Visual appeal***: the overall look and feel of the module	C1	“Again, this is super wordy. I don't know if there's a way to make it more visual.” (P15, clinician-researcher)	Added images and animations to increase visual appeal on text-heavy slides
***Visual assets***: videos, graphics, and animations in the module	C1	“I think that the movements, the videos in the background, are a little bit distracting.” (P8, C1, caregiver) “I don't think it's necessary to have moving images behind a bullet list.” (P20, C1, caregiver)	Replaced animated slide backgrounds with semi-transparent still images to minimize distraction
***Age-appropriateness—design***: age-appropriateness as it relates to the design of the module	C2	“He likes clicking buttons… If you're trying to reach a younger audience, gamifying it a bit would probably be a good idea. I don't think you can have one product both for parents and for kids. I think you absolutely need to create two products. I think the one for kids would be basically a video and a game where you put things in places, and they have a challenge, and it's gamified with a lot of feedback.” (P27, caregiver)	Will consider future adaptation for children
**Learner experience**			
***Engagement***: how engaged users are throughout the module	C1 C2	“I think the requirement to click on the buttons, it seemed kind of annoying, but, at the same time, doing that forces me to focus on the module far more so than if the narrator had just read out every bullet point. That forces me to be engaged much more.” (P17, caregiver) “I find it a little bit frustrating that, as soon as the first [knowledge comprehension] question comes up, I have to wait until the [narrator] reads all of them before I'm allowed to click.” (P21, caregiver)	Increased or decreased the amount of user interaction (i.e., clicks) required to advance the module to optimize user engagement throughout the module Enabled users to instantly interact with the knowledge comprehension questions
***Motivation***: users' motivation to complete the module	C1	“If somebody is looking to get involved, to have already completed this program, and then have it on file themselves. Yes, I think that's a great idea […] I think [a certificate] would be attractive because it gives an element of achievement.” (P5, caregiver) “The only thing I think could make it better is something like a “thank you.” “Congratulations for doing it,” for sure, but also, “thank you for wanting to get engaged in the research process.” (P7, caregiver)	Added completion certificates for each part of Research 101 Added a message to the end of each part thanking viewers for their interest in patient-oriented research
***Length of module***: the length of the module is appropriate	C1	“The page on the benefits of health research with the six points and six check marks, I think that's the same slide that was used at the start of the first part of the module.” (P18, caregiver)	Removed repetition of Benefits of Health Research slide in part 2

*P, participant; C, usability cycle*.

#### Learner-Centered Design

Learner-centered design was divided into subthemes of ease of use, intuitive design, and learnability. Participants described Research 101 as very user-friendly, and most quickly learned how to use the navigational and audio controls. However, in cycle 1 many did not discover the module's interactive features until Part 2. For example:

“I didn't pick up the first time, in Part 1, that the [interactive] menu actually contained an outline of the entire [module].” (participant (P) 3, cycle (C) 1, caregiver)

**Figure 1:**
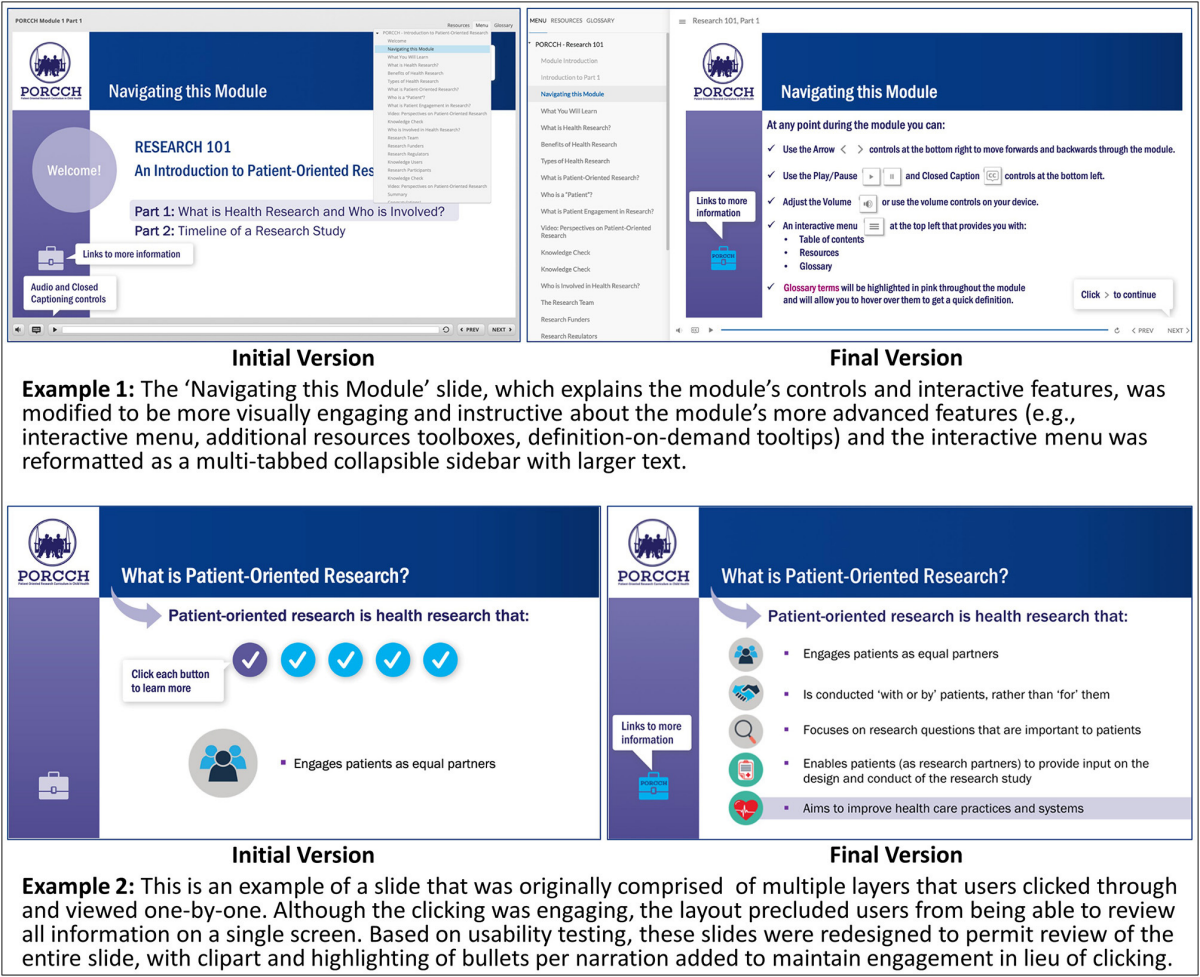
Example modifications made to the e-learning module in response to usability testing.

To make these features more salient, the “Navigating this Module” slide was redesigned to be more attention-grabbing and informative (Figure 1). In addition, participants in cycle 1 found the interactive menu, which was originally formatted as a click dropdown list, difficult to use, as the text was small, and the menu was susceptible to clicking errors. To overcome these issues, the interactive menu was reformatted as a multi-tabbed collapsible sidebar with larger text.

#### Content

Content was evaluated in terms of quantity, completeness, quality and trustworthiness, relevance, usefulness, understandability, and age-appropriateness. Participants across both usability cycles found the information presented in the module to be useful, relevant, and trustworthy:

“I think it does a really good job of two things: encouraging patients and families to get involved because it's emphasizing all the benefits, but it's also being fair about the pitfalls and that there is great potential for delays. So, I thought that was well-balanced.” (P3, C1, caregiver)

Based on feedback in cycle 1, a new slide on potential solutions to the challenges of patient-oriented research was added and positively received by participants in cycle 2. Module sections with poor understandability, because they were too technical, contained undefined terms or concepts, or were considered beyond the scope of an introductory module, were identified in both cycles. To address these issues, instances of jargon were replaced with more common language; key terminology (e.g., authentic partnership) was defined in the glossary; and less relevant sections (e.g., primary versus secondary outcomes) were removed.

#### Aesthetic Design

Within the theme of aesthetic design, subthemes included interactive features, multimedia components, layout, navigation, visual appeal, visual assets, and age-appropriateness. Although participants appreciated having the glossary, some participants in cycle 1 expressed concern that navigating to the glossary might be disruptive or disincentivizing to learners:

“I think the glossary might be lost in translation. I don't know if users are going to click again [to open it], so I don't know if it's useful.” (P7, C1, caregiver)

To address this, glossary definitions were added as tooltips (i.e., text boxes that display information when the cursor is hovered nearby) to all key terms in the module, creating a definition-on-demand feature, using a uniquely colored text to indicate the interactivity. Also, some slides were designed with multiple sequential layers. Participants in cycle 1 did not like that this design precluded them from simultaneously reviewing all content on a single screen; therefore, these slides were redesigned (Figure 1).

Participants liked the module's aesthetic, describing it as inviting and professional-looking, although a lack of diversity in the stock images was noted in cycle 1. In response, the image set was updated to better represent the variety of cultures, age groups, and types of intellectual and physical abilities of those involved in patient-oriented child health research, and a video featuring a discussion between a parent partner and a researcher was replaced with a video from a youth's perspective on being a research partner.

#### Learner Experience

Lastly, learner experience included satisfaction, engagement, memorability, motivation, and length of module. In general, participants in both cycles were highly satisfied with Research 101, finding it engaging and educational:

“It's excellent and I think it's going to be hugely helpful in fast-tracking families who want to get involved in research.” (P4, C1, caregiver)

Most participants regarded the anecdotal videos as the most memorable part of the module. Participants felt the module was an appropriate length but advised that future users consider viewing Parts 1 and 2 spaced apart to maximize focus, engagement, and learning.

### Errors

Errors identified during testing included navigation, audio, presentation, and language errors. Several minor module errors were identified in testing and subsequently fixed, including audio tracks not initiating, multiple audio tracks playing simultaneously, and video buffering issues. In cycle 2, a programming error was unintentionally introduced that resulted in several “dead ends” in the module that compromised module usability.

### System Usability Scale

SUS scores corresponded to ratings of “good” or better in each round. As expected, scores in cycle 2 (75.17 ± 18.14) were lower than cycle 1 (88.33 ± 9.76), given the aforementioned programming error. Cycle 3 scores (87.17 ± 9.95) were consistent with cycle 1, corresponding to an overall usability rating of “excellent.” SUS scores by usability testing cycle and role are presented in Supplementary Appendix 9.

### E-Learning Satisfaction

Overall, learner satisfaction with Research 101 was very high across all 3 cycles (cycle 1: 4.53 ± 0.64; cycle 2: 4.40 ± 0.91; cycle 3: 4.47 ± 0.64), indicating users were generally “very satisfied” with the module. E-learning evaluation results are displayed in Supplementary Appendix 10.

### Evaluation of Impact (Knowledge and Self-Efficacy)

In each round, participants' knowledge test scores, self-reported knowledge of patient-oriented research, and self-efficacy to engage in patient-oriented research increased significantly after completing Research 101 (*p* < 0.01; Table 3).

**Table 3:** Differences in knowledge of and self-efficacy to engage in patient-oriented research before and after completing Research 101.

**Outcome**	**Pre-module (Mean ±SD)**	**Post-module (Mean ±SD)**	**Difference (Mean ±SD)**	***P***
**Self-reported knowledge^a^**				
Cycle 1	3.07 ± 1.39	4.33 ± 0.62	1.27 ± 1.53	**<0.01**
Cycle 2	3.00 ± 0.93	4.13 ± 0.92	1.13 ± 1.06	**<0.001**
Cycle 3	2.80 ± 1.15	4.00 ± 0.65	1.20 ± 1.01	**<0.001**
**Knowledge test** ^b^				
Cycle 1	15.60 ± 2.10	16.93 ± 0.96	1.33 ± 1.72	**<0.01**
Cycle 2	15.00 ± 1.60	16.80 ± 1.37	1.80 ± 1.82	**<0.01**
Cycle 3	15.60 ± 1.68	17.27 ± 0.88	1.67 ± 1.63	**<0.01**
**Self-efficacy** ^c^				
Cycle 1	72.75 ± 17.11	94.11 ± 6.97	21.36 ± 18.27	**<0.001**
Cycle 2	72.29 ± 20.62	87.53 ± 14.85	15.25 ± 14.37	**<0.01**
Cycle 3	68.80 ± 22.54	89.24 ± 11.63	20.44 ± 18.17	**<0.001**

a *Rated on a 1 (I do not know anything about patient-oriented child health research) to 5 (extremely knowledgeable) Likert-type scale*.

b *Possible scores range from 0 to 18, with higher scores indicating greater knowledge of patient-oriented research*.

c *Possible scores range from 0 to 100, with higher scores indicating greater self-efficacy for patient engagement*.

## Interpretation

This paper describes the co-development and evaluation of an e-learning module, “Research 101,” designed to increase the “patient-oriented child health research readiness” of patients and families and other stakeholders without a formal research background. Research 101 was co-developed at all stages with patients and families so that their perspectives, values, and training needs were captured, and end-user usability testing was employed to maximize the usefulness and quality of the module. In each testing cycle, valuable refinements were identified from qualitative and quantitative module evaluations and subsequently implemented. The overall usability of the final version of Research 101 was “excellent,” and the module was shown to significantly improve participants' knowledge of patient-oriented research and self-efficacy to engage in patient-oriented research. As of December 2021, 1 year after launch, PORCCH has had over 50,000 unique website visitors (www.porcch.ca), with over 350 users enrolled in Research 101.

Capacity-building is a key element of SPOR (13) and other national patient and public involvement frameworks, such as the Patient-Centered Outcomes Research Institute (PCORI) (64) in the United States and INVOLVE in the United Kingdom (12). Training of patient and family partners, however, is often variable (30, 65), and typically provided in-person to prepare partners for a specific project (66, 67). Online resources may be a cost-effective way to scale up patient-oriented research training to build capacity and broaden the dissemination of training materials. For example, the online content of the European Patients' Academy on Therapeutic Innovation (EUPATI) Patient Expert Training Programme is being adapted into a massive open online course (MOOC) format to make it more accessible (68). Another example is KidneyPRO, a web-based training module designed for patients and families to promote patient-oriented kidney research in Canada (69). Although there exists broad consensus that lived experience is the most important quality patient partners bring to a research team, there is disagreement on whether and to what extent patient partners should be “further trained” (15, 70). Patient and family partners should take the lead in determining their training needs within the context of the project, and self-guided resources like Research 101 may be helpful in this regard (15).

Several study limitations should be noted. Although the sample size employed was in accordance with recommendations from the usability testing literature (51, 52), it was too small to assess the impact of participant characteristics on module usability. In addition, the usability testers, who were recruited through established family advisory and pediatric research networks for convenience and their familiarity with patient-oriented research, may not fully represent the intended end-users of Research 101. This is a notable limitation, since design processes that do not meaningfully incorporate the lived experience of underrepresented groups can result in products that create barriers for people with a wide range of abilities and backgrounds (71). Best practice in inclusive, accessible, and diverse design is to design with, rather than for, diverse and often excluded communities (71). The self-report measures employed to evaluate the impact of the module on self-efficacy were selected for pragmatic reasons. Evaluation of longer-term outcomes, such as whether completion of Research 101 is associated with increased or more impactful involvement in research, in a larger and more diverse group of patients and families (e.g., with representation of First Nations and other countries) is needed. Lastly, PORCCH is currently only available in English, although a French translation is underway.

### Team Reflections on Patient Engagement

Although the team did not formally collect data to evaluate the process of co-developing Research 101, the team did critically reflect on the processes and outcomes of co-development. General themes that emerged from discussions amongst all team members included the value-add of parent and researcher collaboration, the importance of a broad range of perspectives, the benefits of building relationships and networks, the importance of accessible materials, and the significant time commitment required to ensure authentic partnership. Similar themes have been found in other curriculum-building initiatives (72).

Parents on the steering committee provided valuable input on issues such as accessibility, inclusiveness, and how to achieve authentic and meaningful partnerships. Throughout module testing, they reviewed emerging themes and helped interpret the findings. As noted elsewhere (65, 73), partnering with patients and families was associated with incremental research costs (e.g., compensation for partners) and logistical challenges (e.g., evening meetings to accommodate patient partners). However, the value-add of co-developing the module justified additional budgeting for engagement-related costs. All partners were selected from established children's hospital family advisory networks and were already proficient at collaborating with child health clinicians and researchers. Selection of experienced partners permitted quick initiation of module co-development, but underrepresentation of partners with little or no previous engagement may have contributed in part to an initial module prototype that assumed too much prior knowledge, as revealed by usability testing. To counterbalance this, patients and families with little or no previous engagement were deliberately sought as testers to refine the module and ensure it was targeted appropriately. Having parent and researcher co-leads worked well as perspectives were balanced and the setup provided the parent partner with a dedicated team member for support. The steering committee helped incorporate additional perspectives into the module.

## Conclusions

In conclusion, Research 101, part of the open-access online Patient-Oriented Research Curriculum in Child Health (PORCCH; www.porcch.ca), may be of use to a variety of patients and families and other stakeholders looking for an interactive, introductory curriculum on patient-oriented child health research. Additionally, the methods and findings of this study may help inform the integration of co-development and usability testing in creating other capacity-building resources for patient-oriented research.

## Data Availability Statement

The raw data supporting the conclusions of this article will be made available by the authors, without undue reservation.

## Ethics Statement

The studies involving human participants were reviewed and approved by SickKids Research Ethics Board. Written informed consent to participate in this study was provided by participants, or, where applicable, the participants' legal guardian/next of kin.

## Author Contributions

CW, CM, NJ, and AK: study conception and design. VC and GM: data acquisition. CW, CM, NJ, GM, AK, VC, LP, and AC: analysis, interpretation of data, critical revision, and final manuscript approval. CW, CM, GM, and VC: drafting of the manuscript. All authors contributed to the article and approved the submitted version.

## Funding

This study was funded by a Canadian Institutes of Health Research Strategy for Patient-Oriented Research (SPOR)—Patient-Oriented Research Collaboration Grant #397481 (matching funds were provided by CHILD-BRIGHT, the BC SUPPORT Unit, the Canadian Child Health Clinician Scientist Program, the Ontario Child Health SUPPORT Unit, and the SickKids Research Institute). CW holds an Early Researcher Award from the Ontario Ministry of Research and Innovation. The funder had no role in the design and conduct of the study, decision to publish, or preparation, review, or approval of the manuscript.

## Conflict of Interest

The authors declare that the research was conducted in the absence of any commercial or financial relationships that could be construed as a potential conflict of interest.

## Publisher's Note

All claims expressed in this article are solely those of the authors and do not necessarily represent those of their affiliated organizations, or those of the publisher, the editors and the reviewers. Any product that may be evaluated in this article, or claim that may be made by its manufacturer, is not guaranteed or endorsed by the publisher.
