# Supplementary material for: Co-development and Usability Testing of Research 101: A Patient-Oriented Research Curriculum in Child Health (PORCCH) E-Learning Module for Patients and Families
Source: Front Pediatr. 2022 Jul 6;10:849959. doi: 10.3389/fped.2022.849959 (PMC9297034; doi:10.3389/fped.2022.849959)
Supplement: Supplementary file 1 [file Data_Sheet_1.pdf]

Supplementary Material

Appendix 1: Schematic of PORCCH Module Co-Development Process 2
Appendix 2: Baseline Questionnaire 3
Appendix 3: Usability Testing Questions 5
Appendix 4: Post-Module Interview Guide 6
Appendix 5: E-Learning Satisfaction Questionnaire 9
Appendix 6: Self-Efficacy Questionnaire 10
Appendix 7: Knowledge Test 11
Appendix 8: Qualitative Analysis Coding Framework 13
Appendix 9: SUS Scores by Usability Testing Cycle and Role 15
Appendix 10: E-Learning Module Feedback 16

Appendix 1: Schematic of PORCCH Module Co-Development Process

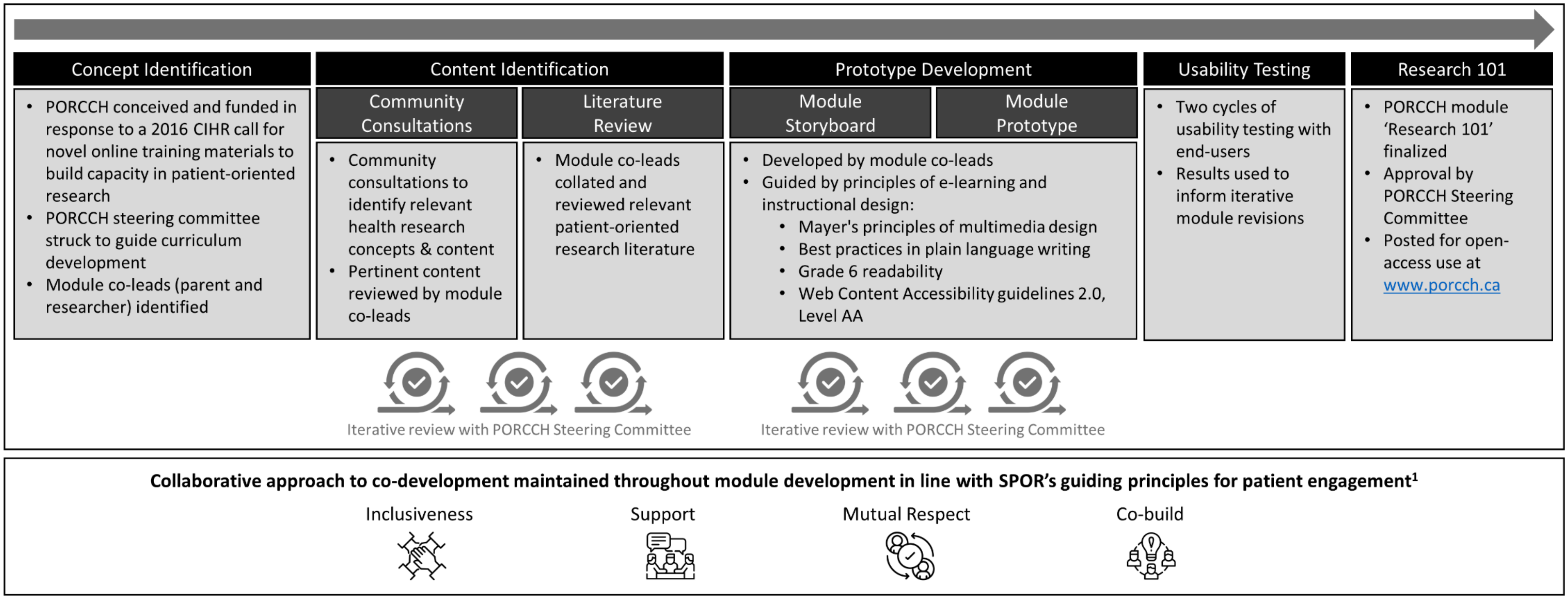

CIHR: Canadian Institutes for Health Research; PORCCH: Patient-Oriented Research Curriculum in Child Health; SPOR: Strategy for Patient-Oriented Research.

References

1. Canadian Institutes of Health Research. Strategy for patient-oriented research: patient engagement framework (2015). Available from: https://cihr-irsc.gc.ca/e/48413.html [Accessed April 8, 2022]

Appendix 2: Baseline Questionnaire

Baseline Questionnaire

1. You are a: ☐ Child ☐ Caregiver ☐ Child health clinician ☐ Researcher ☐ Trainee
2. Age (if < 25 years old): __________
3. Sex: ☐ Male ☐ Female
4. Institution and/or City in which you live: __________________
5. Self-reported Ethnicity:
   a. Country where you were born: _______________
   b. Ethnicity: ☐ Caucasian ☐ Asian ☐ Indian ☐ Other: _____________________
6. List the languages you are able to understand, speak, read and/or write. For each language rate your ability on a 1 (basic ability) to 5 (fluent) scale:

**Language** *(please list)*	**Understanding** *(circle one)*	**Speaking** *(circle one)*	**Reading** *(circle one)*	**Write** *(circle one)*
**English**	**1 2 3 4 5**	**1 2 3 4 5**	**1 2 3 4 5**	**1 2 3 4 5**
	**1 2 3 4 5**	**1 2 3 4 5**	**1 2 3 4 5**	**1 2 3 4 5**
	**1 2 3 4 5**	**1 2 3 4 5**	**1 2 3 4 5**	**1 2 3 4 5**
	**1 2 3 4 5**	**1 2 3 4 5**	**1 2 3 4 5**	**1 2 3 4 5**

7. Please check your ***highest*** education level (or the approximate equivalent to a degree obtained in another country):
   ☐ Elementary school (specify grade: ___________________________)
   ☐ Less than high school (specify grade: _________________________)
   ☐ High School
   ☐ Professional training
   ☐ Some college or university
   ☐ College
   ☐ University
   ☐ Masters
   ☐ MD, PhD, JD
   ☐ Other (please specify: _____________________________________)

9. Profession (if applicable): ____
10. Have you engaged in patient-oriented research previously? ☐ Yes ☐No
    If yes, please explain nature of engagement: ____
    ____
11. Please rate your knowledge regarding patient-oriented research:
    ☐ 1 (I don't know anything about patient oriented research) ☐ 2 ☐ 3 ☐ 4 ☐ 5 (extremely knowledgeable)

12. Have you used e-learning before (online interactive learning?) ☐ Yes ☐ No

13. Please rate your comfort in using a computer:

☐ 1 (don't know how to use a computer) ☐ 2 ☐ 3 ☐ 4 ☐ 5 (extremely comfortable)

14. Please rate your comfort in using the internet:

☐ 1 (don't know how to use a computer) ☐ 2 ☐ 3 ☐ 4 ☐ 5 (extremely comfortable)

Appendix 3: Usability Testing Questions

Questions During Module Testing

Intro script: Now you will go through the modules at your own pace. Please stop at any time and let me know if something grabs your attention either because you like it or dislike it, because you find it is confusing or for any other reason. I will also be stopping you at certain points to ask you questions in order to help us understand how we can make the module better. Once you have completed the module, I will ask you some questions about your feedback. The main purpose of this study is to make these learning modules as useful and user friendly as possible, so we are open to as much feedback as you can provide.

Interviewer notes: Ask participants a number of direct questions at different time points during the module to explore and test participants' understanding of various aspects of the module and to solicit participants' suggestions for improvement. Examples include:

- What do you think of the pictures (graphics)? **(Aesthetics)**
- What do you think of the font, the size of the font, the colour of the font? **(Aesthetics)**
- What do you think of the layout of this slide? **(Aesthetics)**
- How did you find it reading the information on this slide? **(Aesthetics, Content, Ease of Understanding)**
- Where do you think you have to click next? **(Intuitive design, Learnability, Ease of use, Gaps in functionality)**
- What is the module asking you to do at this point? **(Intuitive design)**
- What do you expect to happen? **(Intuitive design)**
- Is it clear whether you need to click (X) vs. (XX), like on this slide? **(Ease of use)**
- What other things, if any, would you want to see or be able to do from this screen? **(Gaps in information)**

Appendix 4: Post-Module Interview Guide

Post-Module Interview Guide

You do not have to ask a question if it was addressed during module completion

- Was the information provided in the modules helpful to you? **(Usefulness)**
  - *What did you find the most useful?*
  - *What did you find least useful?*

- Was there any information that you thought should be in the module but was not there? **(Gaps in information)**
  - *Is there anything you would suggest to add to the e-learning module to make it better?*
  - *Was there any information missing from the module?*

- How much did the module keep your attention as you were going through it? **(Engagement)**
  - *What parts did you pay the most attention to, and why?*
  - *What parts did you pay the least attention to, and why?*

- What parts of the module do you remember the most? **(Memorability)**
  - *What is the main thing you remember about the module?*
  - *What is the main thing you learned that stands out?*

- What parts of the module do you remember the least? **(Memorability)**
  - *Is there anything you are now more confused about?*

- Can you tell me what you liked best about the module? **(Satisfaction, Liked or disliked)**
  - *Information, layout, animations, graphics, etc.?*
  - *Can you tell more about that?*

- Can you tell me what you liked least about the module? **(Satisfaction, Liked or disliked)**
  - *Information, layout, animations, graphics etc.?*
  - *Can you tell more about that?*

- What did you think about the length of the module? **(Efficiency of use)**
  - *Do you think the module should be longer, shorter, or was it just right?*
  - *Why do you think so?*

- Can you tell me about how easy it was to navigate or our find your way around the module? **(Learnability, Ease of use, Gaps in functionality)**
  - *What were the challenges of navigating through the module?*
  - *What would make it easier to navigate through the module?*
  - *What changes would make the module easier to use?*
  - *Were there sections of the e-learning module that you found particularly easy to use?*
    - *What about them made them easy to use?*
  - *Were there sections of the e-learning module that you found particularly hard to use?*
    - *What about them made them hard to use*?

- Can you tell me about what you thought about the overall look of the module? **(Aesthetics)**
  - *For example, the design, colours, and images in the module.*
  - *Does the module feel warm and friendly or cold and technical?*
  - *Can you tell me more about that?*
  - *Do you think it is visually appealing?*
  - *What would make the module more visually appealing?*

- Was the module easy to understand? **(Ease of understanding)**
  - *What did you find easy to understand?*
  - *What did you find difficult to understand?*

- Was the content easy to read? **(Aesthetics, Ease of understanding)**
  - *What did you think of the font?*
  - *What did you think of the amount of text?*

- Did the person talking present information too quickly, or too slowly, or was it just right? **(Ease of understanding)**
  - *What did you think about the pace of the module?*
  - *Were you able to understand what the speaker was saying?*

- Can you tell me what you thought about the information provided on the module? **(Information, Content, Learnability)**
  - *Was the information in the module clearly presented?*
  - *Was it easy to follow along with the audio and writing together?*

- What do you think about the amount of information that was provided? **(Gaps in information)**
  - *Was there too much information? Too little? Or was it just right?*
  - *How much of the information presented in the module did you already know before completing these modules?*

- How do you feel about the accuracy of the information? **(Trustworthiness of information)**
  - *How much do you believe the information provided is true?*
  - *Is there anything you disagree with? Can you specify what/where?*

- If you could make changes to the module, what changes would you make? **(Would like to change/add)**
  - *Can you tell me more about that?*
  - *If you look through these modules (print copy) does anything come to mind?*

- What other things could help you to support what you learned through the module **(Gaps in information)**
  - *Do you think a handout would be helpful? A website? An app?*

- Overall, how satisfied were you with the modules? **(Subjective satisfaction)**
    - *Is there anything you would suggest changing to make the e-learning module better?*

- Can you tell me about whether or not you would use this module to learn more about patient-oriented child health research? **(Usefulness)**
    - *What section of the module would you refer back to, if any?*

- What would motivate you to use the module? **(Motivation)**

- Would you access this module from home if it was made available? **(Motivation)**

- Can you tell me about whether or not you think others would be interested in using this module? **(Usefulness)**
    - *Who would this be most useful for?*
    - *Would you recommend this module others? If so, who? Can you tell me more about that?*

- What would make it easier to motivate others to use the module? **(Motivation)**
    - *What would interest them in using this program?*

- Is there anything else you would like to tell us about the module?
    - *Can you tell me more about that?*

Appendix 5: E-Learning Satisfaction Questionnaire

E-Learning Satisfaction Questionnaire

With regard to the E-Learning Education you just received, please indicate your level of agreement with each question below by marking a number between 1 (low agreement) and 5 (high agreement)

	Low agreement				High agreement
I learned something new	☐ 1	☐ 2	☐ 3	☐ 4	☐ 5
The information I received was easy to understand	☐ 1	☐ 2	☐ 3	☐ 4	☐ 5
I received the right amount of information	☐ 1	☐ 2	☐ 3	☐ 4	☐ 5
My questions were answered	☐ 1	☐ 2	☐ 3	☐ 4	☐ 5
The goals of the session were clear	☐ 1	☐ 2	☐ 3	☐ 4	☐ 5
The length of time it took to finish the e-learning module was good	☐ 1	☐ 2	☐ 3	☐ 4	☐ 5
The e-learning module was easy to use	☐ 1	☐ 2	☐ 3	☐ 4	☐ 5

OVERALL

Please indicate your level of agreement with each question below by marking a number between 1 (not at all) and 5 (very)

	Not at all				Very
Overall, how SATISFIED were you with your education?	☐ 1	☐ 2	☐ 3	☐ 4	☐ 5
Overall, how ENJOYABLE was your education?	☐ 1	☐ 2	☐ 3	☐ 4	☐ 5

Appendix 6: Self-Efficacy Questionnaire

Self-Efficacy Questionnaire

Please rate how certain you are that you understand the following aspects of patient-oriented health research?

Rate your degree of confidence by recording a number from 0 to 100 using the scale given below:

0	10	20	30	40	50	60	70	80	90	100
Cannot do at all				Moderately can do					Highly certain can do	

	Confidence (0 – 100)
Research 101, Part 1: What is Health Research and Who is Involved?	
1. I understand what health research is	_______
2. I understand what patient-oriented child health research is	_______
3. I understand what patient/family engagement in research is	_______
4. I understand the different types of health research	_______
5. I understand who can be involved in health research	_______
Research 101, Part 2: Timeline of a Research Study	
1. I am familiar with the key steps of a research study	_______
2. I understand how patients/families can successfully partner in each step of the research study	_______
3. I understand the types of impact patient-oriented child health research can have	_______
4. I can describe some benefits to patient-oriented child health research	_______
5. I can describe some challenges of patient-oriented child health research	_______

Appendix 7: Knowledge Test

Knowledge Test

Research 101, Part 1: What is Health Research and Who is Involved?

Please circle the answer you believe is correct for each of the following questions:

1)	There are many types of health research from studying cells to studying entire populations	**TRUE**	**FALSE**
2)	Patient and families can engage in all types of health research	**TRUE**	**FALSE**
3)	“Patients" can also refer to parents, siblings, and friends	**TRUE**	**FALSE**
4)	Patient-oriented child health research is related to the recruitment of patients as research subjects	**TRUE**	**FALSE**
5)	Patients and families must provide consent to be a “subject” in a research study	**TRUE**	**FALSE**
6)	The principal investigator is responsible for all aspects of the study	**TRUE**	**FALSE**
7)	A person can be a research subject and a research partner in the same study	**TRUE**	**FALSE**
8)	Patients and families are important knowledge users	**TRUE**	**FALSE**
9)	Knowledge users should only be involved at the end of the study	**TRUE**	**FALSE**

Knowledge Test

Research 101, Part 2: Timeline of a Research Study

Please indicate whether patients and families can partner with researchers to strengthen patient-oriented research in the following ways:

1)	Identify what research questions are important and meaningful to patients and families based on their lived experience	**TRUE**	**FALSE**
2)	Participate in choosing a primary outcome measure that is meaningful to patients and families	**TRUE**	**FALSE**
3)	Participate in designing the study methods	**TRUE**	**FALSE**
4)	Participate in choosing and using patient and family-friendly materials and processes to ensure that collection of information is made as easy and efficient as possible	**TRUE**	**FALSE**
5)	Suggesting ways to recruit subjects	**TRUE**	**FALSE**
6)	Interpreting the results of the study from a patient and family perspective	**TRUE**	**FALSE**

Please circle the answer you believe is correct for each of the following questions:

1.	Engaging in patient-oriented research can help you gain research experience and skills	**TRUE**	**FALSE**
2.	There can be costs associated with being a research partner	**TRUE**	**FALSE**
3.	Research studies are often completed quickly	**TRUE**	**FALSE**

Appendix 8: Qualitative Analysis Coding Framework

*Denotes codes that were created and defined during the coding process, versus established usability attributes or subthemes from previous studies

Learner-centered design

- **Ease of use**: How users perceive the ease of use and functionality of the module (1–3)
- **Intuitive design**: The ease at which users know what to do next (e.g., all prompts; toolbox–prompt needed) (2,3)
- **Learnability**: Capability of the module to teach users how to use it (1–7)

Content

- **Quantity**: Amount of information (2)
- **Completeness**: The extent to which the module content contained all desired information (3,5)
- **Quality and trustworthiness**: The extent to which users perceived the content to be accurate and credible (3)
- **Age-appropriateness – content**: As it relates to the content presented in the module (3)
- **Relevance**: Relevance to a key group in child health research (how or why) (2,6)
- **Understandability**: Content aspects such as readability, use of plain/lay language, and explanation of medical terminology (3,4,6)
- **Usefulness**: How useful the information was or who the information would be useful for (3,6)

Aesthetic design

- **Multimedia components**: Audio and visual aspects of the module not covered under visual assets (e.g., narration, slides advancing too quickly) (3,5,6)
- **Features**: Technical/interactive elements of module (e.g., knowledge check questions, hover/glossary, highlighting) (3,6)
- **Layout**: Spacing/the arrangement of text/graphics/etc. on a slide (e.g., font size readability) (2)
- **Age-appropriateness – design**: As it relates to aesthetic design (3)
- **Navigation**: The ability of the user to easily move around the module (e.g., is user repeating/jumping/skipping ahead/skipping through?) (2–6)
- **Visual appeal**: The overall look and feel of the module (2–6)
- **Visual assets**: Videos, illustrations, graphics, and animations in the module (3)

Learner experience

- **Motivation**: Motivation to complete the module (6)
- **Engagement**: How engaged users are as they proceed through the module (5,6)
- **Memorability**: Ease of remembering module information (e.g., information or aspect of the module that really stuck out) (3,4,7)
- **Satisfaction**: User satisfaction with the module (1,3,4,6,7)
- **Length of module***: Is it too long? Too short? Just right?

Errors

- **Navigation error**: User didn't navigate through a part of the module in the correct order (3, 7)
- **Language***: Any language-related error in the module
- **Audio**: Audio-related errors (text-audio mismatches, volume inconsistencies/problems) (3)
- **Presentation error**: Technical problems with the module that aren't audio- or language-related (6)

Categorization

- **Positive***
- **Negative***
- **Ambivalent***
- **Suggested change***

References

1. Shackel B. Usability – context, framework, design and evaluation. In: Shackel B, Richardson SJ, editors. Human Factors for Informatics Usability. Cambridge, UK: Cambridge University Press; 1991. P. 21–38.
2. Koohang A, Paliszkiewicz J. E-Learning courseware usability: building a theoretical model. J Comput Inf Syst. 2015;56(1):55–61. Doi: 10.1080/08874417.2015.11645801
3. Connan V, Marcon MA, Mahmud FH, Assor E, Martincevic I, Bandsma RH, et al. Online education for gluten-free diet teaching: development and usability testing of an e-learning module for children with concurrent celiac disease and type 1 diabetes. Pediatr Diabetes. 2019;20(3):293–303. Doi: 10.1111/pedi.12815
4. Koohang A, Du Plessis J. Architecting usability properties in the e-learning instructional design process. Int J E-learning. 2004;3(3):38–44.
5. Sandars J, Lafferty N. Twelve tips on usability testing to develop effective e-learning in medical education. Med Teach. 2010;32(12):956–60. Doi: 10.3109/0142159X.2010.507709
6. Zaharias, P, Poylymenakou, A. Developing a usability evaluation method for e-learning applications: beyond functional usability. Int J Hum-Comput Int. 2009;25(1):75–98. Doi: 10.1080/10447310802546716
7. Nielsen J. Usability engineering. San Diego, CA: Academic Press; 1993.

Appendix 9: System Usability Sscale Scores by Usability Testing Cycle and Role

	N	Mean (SD)	Min–Max
Cycle 1			
Patients	2	92.50 (7.07)	87.5–97.5
Caregivers	11	89.09 (9.50)	70–100
Clinician-researchers	2	80.00 (14.14)	70–90
Cycle 2			
Patients	2	65.00 (28.28)	45–85
Caregivers	11	77.73 (18.01)	42.5–100
Clinician-researchers	2	71.25 (15.91)	60–82.5
Cycle 3			
Patients	2	88.75 (12.37)	80–97.5
Caregivers	10	85.00 (9.93)	70–100
Clinician-researchers	3	93.33 (9.46)	82.5–100

SD: Standard deviation

Appendix 10: E-Learning Module Feedback

Question[a]	**Cycle 1** (Mean ± SD) n = 15	**Cycle 2** (Mean ± SD) n = 15	**Cycle 3** (Mean ± SD) n = 15
I learned something new	4.40 ± 0.74	4.27 ± 0.96	4.60 ± 0.63
The information I received was easy to understand	4.53 ± 0.64	4.27 ± 1.03	4.80 ± 0.41
I received the right amount of information	4.80 ± 0.41	4.20 ± 1.08	4.50 ± 0.76
My questions were answered	4.67 ± 0.49	4.27 ± 0.96	4.33 ± 0.82
The goals of the session were clear	4.73 ± 0.59	4.33 ± 1.05	4.73 ± 0.59
The length of time it took to finish the e-learning modules was good	4.67 ± 0.49	3.73 ± 1.28	4.33 ± 0.98
The e-learning module was easy to use	4.73 ± 0.46	4.13 ± 0.92	4.67 ± 0.62
Overall, how SATISFIED were you with your education?[b]	4.53 ± 0.64	4.40 ± 0.91	4.47 ± 0.64
Overall, how ENJOYABLE was your education?[b]	4.47 ± 0.92	4.00 ± 1.07	4.13 ± 0.64

SD: Standard deviation
[a]Rated on a 1 (low agreement) to 5 (high agreement) Likert-type scale.
[b]Rated on a 1 (not at all) to 5 (very) Likert-type scale.
